# ID 217 - Modelo Preditivo para Internações por Doenças Respiratórias e sua Sazonalidade, no Contexto Pós-Pandêmico: estudo de série temporal

**DOI:** 10.5327/2237-9622.2025.v34s1.217

**Published:** 2025-11-25

**Authors:** Marcus Carvalho Borin, Ana Paula Serra, Fernando Martin Biscione, Carina Rejane Martins, Daniel Pitchon dos Reis, Geraldo José Coelho Ribeiro, Júlia Teixeira Tupinambás, Karina de Castro Zocrato, Lélia Maria de Almeida Carvalho, Marcela Pinto de Freitas, Maria da Glória Cruvinel Horta, Mariana Michel Barbosa, Mariza Cristina Torres Talim, Sergio Adriano Loureiro Bersan, Silvana Marcia Bruschi Kelles

**Keywords:** modelo preditivo, doenças respiratórias, covid-19, previsão de internações, Unimed-BH

## Abstract

**Introdução:**

A pandemia de covid-19 impactou os padrões de internação por doenças respiratórias, alterando o perfil sazonal tradicionalmente observado em populações mais vulneráveis, como crianças e idosos. Este estudo tem como objetivo desenvolver um modelo preditivo capaz de antever a demanda por internações hospitalares por doenças respiratórias no período pós-pandêmico, utilizando dados da Unimed-BH, uma das maiores cooperativas de saúde do Brasil. O provisionamento de leitos é essencial para a assistência e a alocação de recursos de saúde, especialmente diante das mudanças sazonais causadas pela pandemia.

**Método:**

Foi realizado um estudo retrospectivo de séries temporais, utilizando dados de internação por doenças respiratórias na Unimed-BH, entre 2014 e 2024. Para a previsão de internações no período pós-pandêmico, foi aplicado o modelo STLF ().

**Resultados:**

O modelo STLF apresentou variações no desempenho ao longo dos horizontes temporais de previsão. Para previsões de quatro semanas, o MAPE ( – em português, erro médio percentual absoluto) foi de 10,84%, indicando uma precisão acurada. No entanto, para previsões de 12 semanas, o MAPE aumentou para 28,79%, refletindo a dificuldade de capturar a variabilidade sazonal em médio prazo. As previsões de oito semanas apresentaram um MAPE intermediário de 18,80%. O modelo mostrou-se eficaz para previsões de curto prazo, mas enfrentou limitações em períodos mais longos, devido à volatilidade introduzida pela pandemia de covid-19.

**Conclusão:**

A utilização do modelo STLF para prever internações por doenças respiratórias demonstrou boa acurácia para antecipar a demanda em horizontes de curto prazo, sendo uma ferramenta útil para gestores de saúde no planejamento de recursos hospitalares. No entanto, a precisão diminui em previsões de médio prazo, evidenciando a necessidade de ajustes contínuos nos modelos preditivos para lidar com a incerteza introduzida por eventos como a pandemia. Este estudo reforça a importância de desenvolver modelos adaptativos para prever picos de internação em cenários de alta variabilidade.

